# Preparation and Characterization of Light-Colored Polyimide Nanocomposite Films Derived from a Fluoro-Containing Semi-Alicyclic Polyimide Matrix and Colloidal Silica with Enhanced High-Temperature Dimensionally Stability

**DOI:** 10.3390/polym15143015

**Published:** 2023-07-12

**Authors:** Zhibin He, Xi Ren, Zhenzhong Wang, Zhen Pan, Yuexin Qi, Shujun Han, Haifeng Yu, Jingang Liu

**Affiliations:** 1 School of Material Science and Engineering, Key Laboratory of Polymer Chemistry and Physics of Ministry of Education, Peking University, Beijing 100871, China; zb.he@stu.pku.edu.cn (Z.H.); yuhaifeng@pku.edu.cn (H.Y.); 2 Engineering Research Center of Ministry of Education for Geological Carbon Storage and Low Carbon Utilization of Resources, School of Materials Science and Technology, China University of Geosciences, Beijing 100083, China; renxi@email.cugb.edu.cn (X.R.); wzz0808@163.com (Z.W.); 2103210036@email.cugb.edu.cn (Z.P.); qiyuexin1004@163.com (Y.Q.); 15966200097@163.com (S.H.)

**Keywords:** polyimide film, optical transparency, coefficient of thermal expansion (CTE), benzanilide, thermal properties

## Abstract

Light-colored and transparent polyimide (PI) films with good high-temperature dimensional stability are highly desired for advanced optoelectronic applications. However, in practice, the simultaneous achievement of good optical and thermal properties in one PI film is usually difficult due to the inter-conflicting molecular design of the polymers. In the present work, a series of PI-SiO_2_ nanocomposite films (ABTFCPI) were developed based on the PI matrix derived from hydrogenated pyromellitic anhydride (HPMDA) and an aromatic diamine containing benzanilide and trifluoromethyl substituents in the structure, 2,2′-bis(trifluoromethyl)-4,4′-bis [4-(4-aminobenzamide)]biphenyl (ABTFMB). The inorganic SiO_2_ fillers were incorporated into the nanocomposite films in the form of colloidal nanoparticles dispersed in the good solvent of N,N-dimethylacetamide (DMAc) for the PI matrix. The derived ABTFCPI nanocomposite films showed good film-forming ability, flexible and tough nature, good optical transparency, and good thermal properties with loading amounts of SiO_2_ up to 30 wt% in the system. The ABTFCPI-30 film with a SiO_2_ content of 30 wt% in the film showed an optical transmittance of 79.6% at the wavelength of 400 nm (T_400_) with a thickness of 25 μm, yellow index (b*) of 2.15, and 5% weight loss temperatures (T_5%_) of 491 °C, which are all comparable to those the pristine ABTFCPI-0 matrix without filler (T_400_ = 81.8%; b* = 1.77; T_5%_ = 492 °C). Meanwhile, the ABTFCPI-30 film exhibited obviously enhanced high-temperature dimensional stability with linear coefficients of thermal expansion (CTE) of 25.4 × 10^−6^/K in the temperature range of 50 to 250 °C, which is much lower than that of the AMTFCPI-0 film (CTE = 32.7 × 10^−6^/K).

## 1. Introduction

Colorless or light-colored polyimide (CPI) films have been widely investigated as high-performance components in modern optoelectronic applications due to their excellent combined thermal, optical, dielectric, and other functionalities [1,2,3]. However, the common CPI films, either the fluoro-containing or the semi-alicyclic ones, usually suffer from relatively poor high-temperature dimensional stability; that is, the high linear coefficients of thermal expansion (CTE) compared with the commonly used metal or inorganic materials in the current optoelectronic fabrications. For example, the CPI films derived from fluoro-containing dianhydride, 4,4′-hexafluoroisopropylene diphthalic anhydride (6FDA), and fluoro-containing diamine, 2,2′-bis(trifluoromethyl)benzene (TFMB) showed a CTE value of 82 × 10^−6^/K [4]. The semi-alicyclic CPI films derived from hydrogenated pyromellitic anhydride (HPMDA) and 4,4′-oxydianiline (ODA) had a CTE value of 57.1 × 10^−6^/K [5]. Comparatively, the first generation of silicon-based semiconductor chips showed a CTE value of 3.0 × 10^−6^/K [6], and the third generation of silicon carbide (SiC)-based chips showed a CTE value of 4.2 × 10^−6^/K [7]. The optical sodalime glass (SLG) and the new generation of ultrathin glass (UTG) showed CTE values of 8.9 × 10^−6^/K and 7.2 × 10^−6^/K, respectively [8]. The mismatch between the CTE values of CPI films and the inorganic components might induce internal stress during the fabrication of the optoelectronic devices, which might result in delamination, cracking, warpage, and other reliability issues [9]. Thus, it is becoming one of the most important topics for the improvements of the high-temperature dimensional stability of the CPI films [10,11,12].

Among various CPI films, the ones based on the alicyclic dianhydrides and aromatic diamines, that is, the semi-alicyclic (or semi-aromatic) systems, have attracted great attention in the research and development of high-performance polymers for optoelectronic applications due to the excellent combined thermal, optical, mechanical, and dielectric properties and the relatively low cost at the same time [13,14,15,16]. Various methodologies based on the molecular design of the functional alicyclic dianhydride or the aromatic diamine monomers have been proposed and carried out to reduce the CTE values of the semi-alicyclic CPI films. For instance, an alicyclic dianhydride monomer having a rigid cyclopentanone bis-spironorbornane structure (CpODA) was developed, and the derived CPI films showed reduced CTE values in the range of 17 × 10^−6^/K to 57 × 10^−6^/K [17]. An alicyclic dianhydride, octahydro-2,3,6,7-anthracenetetracarboxylic dianhydride (OHADA) was recently reported, and the CPI films based on the dianhydride monomer showed a CTE value as low as 41.5 × 10^−6^/K [18]. An alicyclic dianhydride monomer containing a rigid cyclobutane unit, 1,3-dimethyl-1,2,3,4-cyclobutane tetracarboxylic dianhydride (DM-CBDA), was synthesized to develop low-CTE CPI films [19]. The CPI film based on DM-CBDA and TFMB exhibited a CTE value of 28.1 × 10^−6^/K, which was much lower than that of the 6FDA-TFMB system (CTE = 82 × 10^−6^/K). For the diamine monomers, various rigid-rod units have been introduced into the molecular structures of the compounds so as to reduce the CTE values of the derived semi-alicyclic CPI films. Amide (-CONH-) or benzanilide (-Ph-CONH-Ph-) structural units have been proven to be the most effective substituents for achieving this target [20,21,22]. In our previous work, the aromatic diamines containing amide linkages were developed, and the CPI films based on the diamines and the hydrogenated pyromellitic anhydride (HPMDA) showed reduced CTE values as low as 27.7 × 10^−6^/K [23]. However, the optical properties of the derived low-CTE CPI films deteriorated with the incorporation of the amide components. In view of this defect of the common CPI films derived from the amide-containing diamines, a novel aromatic diamine simultaneously containing rigid-rod benzanilide and highly electronegative trifluoromethyl (–CF_3_) units, 2,2′-bis (trifluoromethyl)-4,4′-bis[4-(4-aminobenzamide)]biphenyl (ABTFMB) was designed and developed in Hasegawa’s group and was widely used to develop high-performance CPI films [24,25,26].

Although the semi-alicyclic CPI films derived from alicyclic dianhydride and the ABTFMB or its derivative diamines usually represent the high-performance films with both high optical transparency and high thermal stability, it is often required to further decrease the CTE values so as to meet the ever-increasing reliability requirements in the optoelectronic fields. Composite films seem to be one of the few feasible pathways to achieve this target. Combination with inorganic nanofillers with low thermal expansion characteristics, such as silica (SiO_2_), has been proven to be an effective way to further reduce the CTE values of the CPI films [27,28,29,30]. However, the incorporation of nanoparticles might deteriorate the optical transparency of derived composite films. This requires careful design of the morphology, particle size, and surface pretreatment of the nanoparticles. Recently, a special class of silica nanoparticles, colloidal-type silica, has been widely used in the modification of CPI films [29]. In practical applications, colloidal silica (cSiO_2_) nanoparticles are usually dispersed in a good solvent, such as N,N-dimethylacetamide (DMAc), for the CPI film manufacturing, and thus can form uniform dispersion and distribution in the CPI matrix after high-temperature imidization or curing process. The unique physical and chemical properties of the colloidal silica nanoparticles make them achieve a higher loading proportion in the CPI matrix than the traditional nano-silica particles, which can effectively reduce the CTE values of the composite films while maintaining the inherent optical properties of the CPI matrix film to a large extent. Yu and coworkers reported the highly transparent PI/silica hybrid optical thin films based on the wholly aromatic fluoro-containing PI matrix and cSiO_2_ fillers [30,31]. The derived nanocomposite films showed good optical transparency. Although the wholly aromatic fluoro-containing PI films have been widely used as the matrix for the development of the PI/cSiO_2_ nanocomposite films, the CPI/cSiO_2_ films based on fluoro-containing semi-alicyclic PI films have been rarely reported in the literature.

In the current work, a synergistic methodology was used to develop high-performance CPI films with good dimensional stability at elevated temperatures. On the one hand, the semi-alicyclic CPI matrix film (ABTFCPI-0) with the low-CTE feature was prepared from HPMDA dianhydride and ABTFMB diamine. On the other hand, colloidal SiO_2_ nanoparticles were used as the filler to further reduce the CTE values of the ABTFCPI-0 matrix. Effects of the loading amounts of the colloidal SiO_2_ nanoparticles on the properties of the afforded composite films were studied in detail.

## 2. Materials and Methods

### 2.1. Materials

Hydrogenated pyromellitic anhydride (HPMDA, purity: 99.7%) and 2,2′-bis(trifluoromethyl)-4,4′-bis[4-(4-aminobenzamide)]biphenyl (ABTFMB, purity: 99.2%) monomers were all prepared and purified in our laboratory. The alicyclic HPMDA dianhydride and the ABTFMB diamine were dried at 180 °C and 80 °C in vacuum for 24 h, respectively, prior to use. The colloidal SiO_2_ nanoparticles (average diameter: 15 nm; 20 wt% in DMAc) were also prepared in our laboratory by replacing the dispersing agent of pure water of commercially available water-based colloidal SiO_2_ (VPS NE20, Evonik Industrial Co., Ltd., Darmstadt, Germany) with ultra-dry DMAc solvent (InnoChem Science & Technology Co., Ltd., Beijing, China). The ultra-dry γ-butyrolactone (GBL) solvent with water contents below 50 ppm (InnoChem Science & Technology Co., Ltd., Beijing, China), toluene, and other commercially available reagents were used as received.

### 2.2. Characterization Methods

The number average molecular weight (M_n_) and weight average molecular weight (M_w_) of the ABTFCPI-0 resin were measured using a gel permeation chromatography (GPC) system (Shimadzu, Kyoto, Japan) with HPLC grade N-methyl-2-pyrrolidone (NMP) as the mobile phase. Fourier transform infrared (FTIR) spectra of the CPI films were recorded on an Iraffinity-1S FT-IR spectrometer (Shimadzu, Kyoto, Japan). Hydrogen nuclear magnetic resonance (^1^H-NMR) of the ABTFCPI-0 resin was measured on an AV 400 spectrometer (Ettlingen, Germany) operating at 400 MHz in deuterated dimethyl sulfoxide (DMSO-d_6_). Ultraviolet–visible (UV-Vis) spectra of the CPI films were recorded on a Hitachi U-3210 spectrophotometer (Tokyo, Japan) at room temperature. Wide-angle X-ray diffractions (XRD) of the CPI films were performed on a Rigaku D/max-2500 X-ray diffractometer (Tokyo, Japan) with Cu-Kα1 radiation, operated at 40 kV and 200 mA. The full-width at half-maximum (FWHM) values of the ABTFCPI samples were calculated using Scherrer’s equation together with the Jade software [32]. X-ray photoelectron spectroscopy (XPS) was measured with an ESCALab220i-XL electron spectrometer (Thermo Fisher Scientific, Franklin, MA, USA) using 300 W of MgKα radiation. Yellow index (YI) values of the CPI films were measured using an X-rite color i7 spectrophotometer (Grand Rapids, MI, USA) with PI samples at a thickness of 50 μm. The color parameters were recorded according to a CIE Lab equation. L* is the lightness, where 100 means white and 0 implies black. A positive a* means a red color and a negative one indicates a green color. A positive b* means a yellow color and a negative one indicates a blue color. Thermogravimetric analyses (TGA) of the CPI films were recorded on a TA-Q series thermal analysis system (New Castle, DE, USA) at a heating rate of 20 °C/min in nitrogen. Dynamic mechanical analysis (DMA) was recorded on a TA-Q800 thermal analysis system (New Castle, DE, USA) at a heating rate of 5 °C/min and a frequency of 1 Hz in nitrogen. Thermo-mechanical analyses (TMA) of the CPI films were performed on a TMA402F3 thermal analysis system (NETZSCH, Selb, Germany) with a heating rate of 5 °C/min in nitrogen. The CTE values of the CPI films were recorded in the range of 50~250 °C.

### 2.3. CPI Resin Synthesis and the Film Preparation

The ABTFCPI-0 matrix resin was synthesized via a well-established one-stage high-temperature polycondensation procedure in the literature. As a demonstration, an oil bath, Dean–Stark trap, and a nitrogen inlet were added ABTFMB (55.8470 g, 100 mmol) and GBL (200.0 g) into a 1000 mL four-necked glass vessel equipped with a mechanical stirrer. Nitrogen was passed through the diamine solution in the flask, and a transparent ABTFMB solution was obtained after stirring at room temperature for 30 min. HPMDA (22.4170 g, 100 mmol) was then added to the ABTFMB solution, and GBL (34.8 g) was added to make the solid content of the polymerization system to be 25 wt%. The reaction system was stirred for 3 h, and the solution temperature slightly increased. Then, the imidization catalyst of isoquinoline (0.5 g) and the water azeotropic agent of toluene (200 mL) were added. The reaction system was heated via the oil bath, and the toluene–water azeotrope appeared in the system when the temperature reached 130~135 °C. The water by-product was continuously distilled out of the system via the azeotrope until no droplet was observed in the Dean–Stark trap (~6 h). The residual toluene was distilled out of the reaction system, and the temperature gradually increased to 180 °C. The polymerization temperature was maintained for 6 h and then cooled to room temperature. The obtained viscous solution with the pale-brown color was then poured into an aqueous ethanol solution (1000 mL, ethanol:water = 70:30, volume ratio). The off-white silky resin continuously precipitated into the ethanol solution. The obtained ABTFCPI-0 resin was immersed in the ethanol solution for 48 h and then collected and dried at 100 °C in a vacuum for 24 h. Yield: 71.9 g (96.3%). Numeric average molecular weight (M_n_): 1.11 × 10^5^ g/mol; weight average molecular weight (M_w_): 2.08 × 10^5^ g/mol; polydispersity index (PDI): 1.87. ^1^H-NMR (DMSO-d_6_, ppm): 10.77 (s, 2H), 8.37–8.08 (m, 8H), 7.57–7.40 (m, 6H), 3.33–3.30 (m, 4H), and 2.28–2.09 (m, 4H).

The ABTFCPI-0 resin was re-dissolved in ultra-dry DMAc solvent at room temperature according to the formulas shown in Table 1. Then, the colloidal silica (cSiO_2_)/DMAc solution was dispersed into the ABTFCPI-0/DMAc solution under supersonic treatment for 1 h. The total solid contents of the CPI solutions were controlled to be 20 wt%. The weight percent of the colloidal silica nanoparticles in the CPI films increased from 0 (ABTFCPI-0) to 30 wt% (ABTFCPI-30). Taking ABTFCPI-30 as an example, ABTFCPI-0 resin (14.0 g) was added into DMAc (56.0 g) in a 250 mL three-necked flask equipped with a mechanical stirrer. The solution was stirred overnight until a homogeneous and transparent varnish was obtained. The varnish was then purified by filtration through a 0.45 μm Teflon syringe filter. The purified solution was mechanically blended with the colloidal silica dispersion (30.0 g) at room temperature for 2 h and then further treated with a supersonic oscillator for another 1 h. The obtained varnish was coated on a clean borosilicate glass and thermally dried in a clean oven with the heating procedure of 80 °C/3 h, 150 °C/1 h, 180 °C/1 h, 200 °C/1 h, and 250 °C/1 h. After the thermal treatment, the glass substrate was cooled to room temperature and immersed in deionized water. Free-standing ABTFCPI-30 film automatically peeled off the substrate, which was further dried in a vacuum at 120 °C for 24 h. ABTFCPI-0 films with various thicknesses in the range of 10–100 μm were separately prepared for the following characterization. The other composite films, including ABTFCPI-5, ABTFCPI-10, ABTFCPI-15, ABTFCPI-20, and ABTFCPI-25, were prepared according to a similar procedure as mentioned above. For the preparation of the pure ABTFCPI-0 film, no colloidal silica nanoparticles were added.

## 3. Results and Discussion

### 3.1. CPI Resin Synthesis and Film Preparation

**Figure 1:**
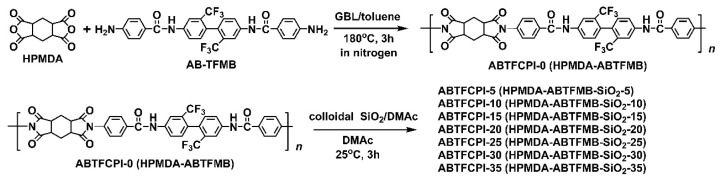
Preparation of semi-alicyclic amide-bridged PIs.

One pure CPI (ABTFCPI-0) and seven CPI nanocomposite films (ABTFCPI-5~ABTFCPI-35) were prepared, respectively, based on the ABTFCPI-0 matrix resin, as shown in Figure 1. The resin was soluble in the polycondensation system despite the existence of the rigid-rod benzanilide units in the diamine moiety. The non-conjugated molecular structure feature in the HPMDA dianhydride moiety endowed the derived resin with good solubility in the reaction media. The resin was soluble in both polar aprotic solvents, such as N,N–dimethylformamide (DMF), DMAc, NMP, dimethyl sulfoxide (DMSO), and common polar solvents, such as cyclopentanone, chloroform, and so on at the solid content of 10 wt%.

**Figure 2:**
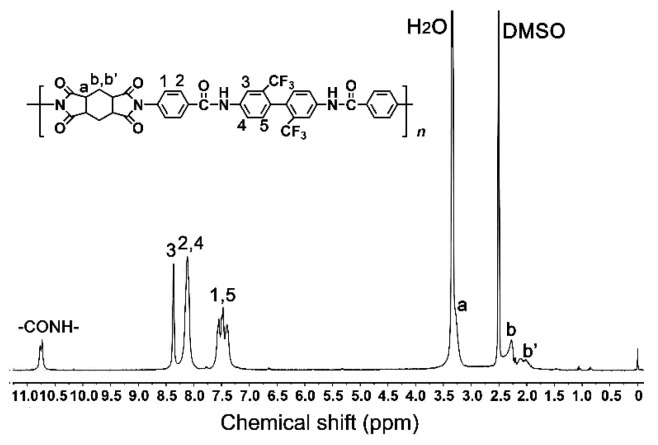
^1^H-NMR spectrum of ABTFCPI-0 resin.

The good solubility of the ABTFCPI-0 resin in organic solvents made it possible to confirm the structure by ^1^H-NMR measurements, as illustrated in Figure 2. In the figure, the characteristic absorptions of hydrogen protons in the amide (–CONH–) units, in the aromatic rings (H_1_~H_5_), and in the alicyclic rings (H_a_, H_b_, and H_b′_) could be clearly assigned. As expected, the amide proton exhibited absorption at the chemical shift of 10.77 ppm, which was at the farthest downfield in the spectra. This is due to the strong electron-withdrawing carbonyl group. Similarly, the absorption of H_3_ appeared at the second farthest downfield in the spectra due to the ortho-substituted –CF_3_ groups with high electronegativity. Contrarily, the absorptions of the cyclohexane protons were present in the upfield in the spectra due to the electron-donating features of the alicyclic rings. This is consistent with the structural features of the ABTFCPI-0 resin.

**Figure 3:**
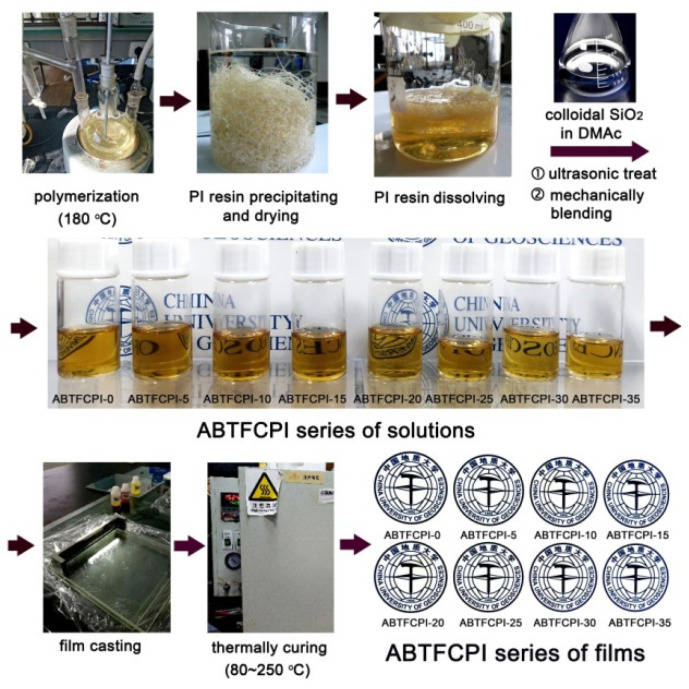
Preparation of ABTFCPI-0 resin and the fabrication diagram of ABTFCPI series of nanocomposite films with the ABTFCPI-0 resin as the matrix and the cSiO_2_ as the fillers.

The pure ABTFCPI-0 film and the ABTFCPI-SiO_2_ nanocomposite films (ABTFCPI-5~ABTFCPI-35) were prepared according to the procedure shown in Figure 3. All the films showed flexible and tough characters except ABTFCPI-35, which was a bit brittle and could not be folded like the other counterparts. The good flexibility of the nanocomposite films was, on the one hand, due to the merits of the ABTFCPI-0 matrix and, on the other hand, owing to the good dispersion and distribution of the SiO_2_ nanoparticles. The ABTFCPI-0 resin showed a numerical average molecular weight (M_n_) of 1.11 × 10^5^ g/mol, average molecular weight (M_w_) of 2.08 × 10^5^ g/mol, and polydispersity index (PDI) of 1.87. The high molecular weights of the resin could endow the derived films with good strength and toughness. As for the colloidal silica nanoparticles, they could be uniformly dispersed into the pristine ABTFCPI-0 matrix due to the specific physical and chemical features of the fillers. Few aggregations were observed for the nanoparticles in the composite films, as could be evidenced by the clear and transparent appearance of the composite solutions and films shown in Figure 3. Even though the loading amounts of the SiO_2_ fillers reached 35 wt%, the composite films still maintain good optical transparency.

**Figure 4:**
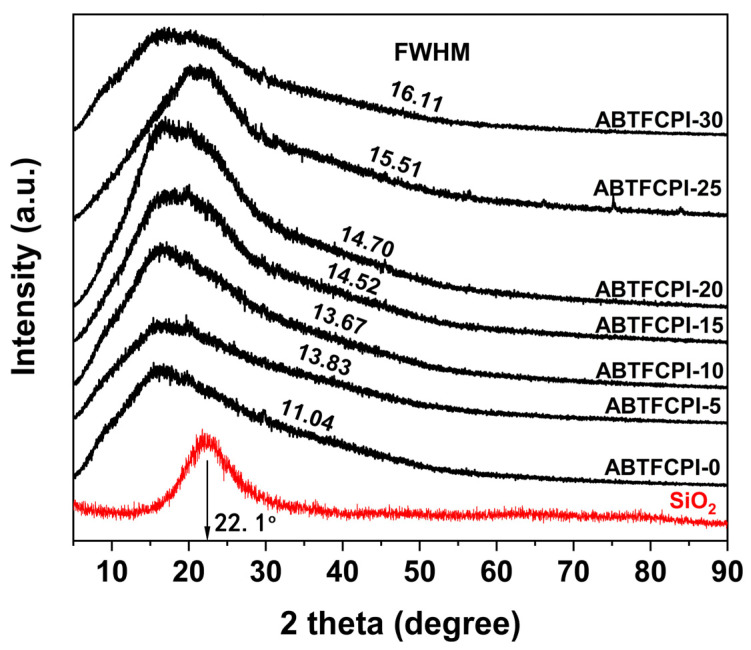
XRD patterns of ABTFCPI films.

The successful dispersion of the SiO_2_ nano-fillers in the composite films could further be proven by the XRD and XPS measurements. As could be seen from the XRD plots of the polymer films shown in Figure 4, the pristine SiO_2_ nanoparticles, the ABTFCPI-0 film, and the nanocomposite films all exhibited amorphous nature. This is mainly ascribed to the non-conjugated feature of the cyclohexane rings in the dianhydride moiety, which efficiently prohibited the formation of crystalline regions in the polymers. The amorphous colloidal silica nanoparticles showed the absorption peak at 2θ = 22.1° (d spacing = 4 Å), which was in good agreement with the data reported in the literature [33]. In addition, it could be deduced from the full width at half maxima (FWHM) values of the samples labeled in the figure that the FWHM values increased with the increasing SiO_2_ contents in the films. This indicates that the partially ordered molecular packing structures in the films were gradually destroyed with the increasing contents of the SiO_2_. On the other hand, it also indicates that with the increase of the SiO_2_ fillers, some interactions between the SiO_2_ particles and the ABTFCPI-0 matrix occurred, causing a lower crystallinity in the films. The loose molecular packing in the nanocomposite films was beneficial for the penetration of visible light and endowed the films with good optical transparency.

**Figure 5:**
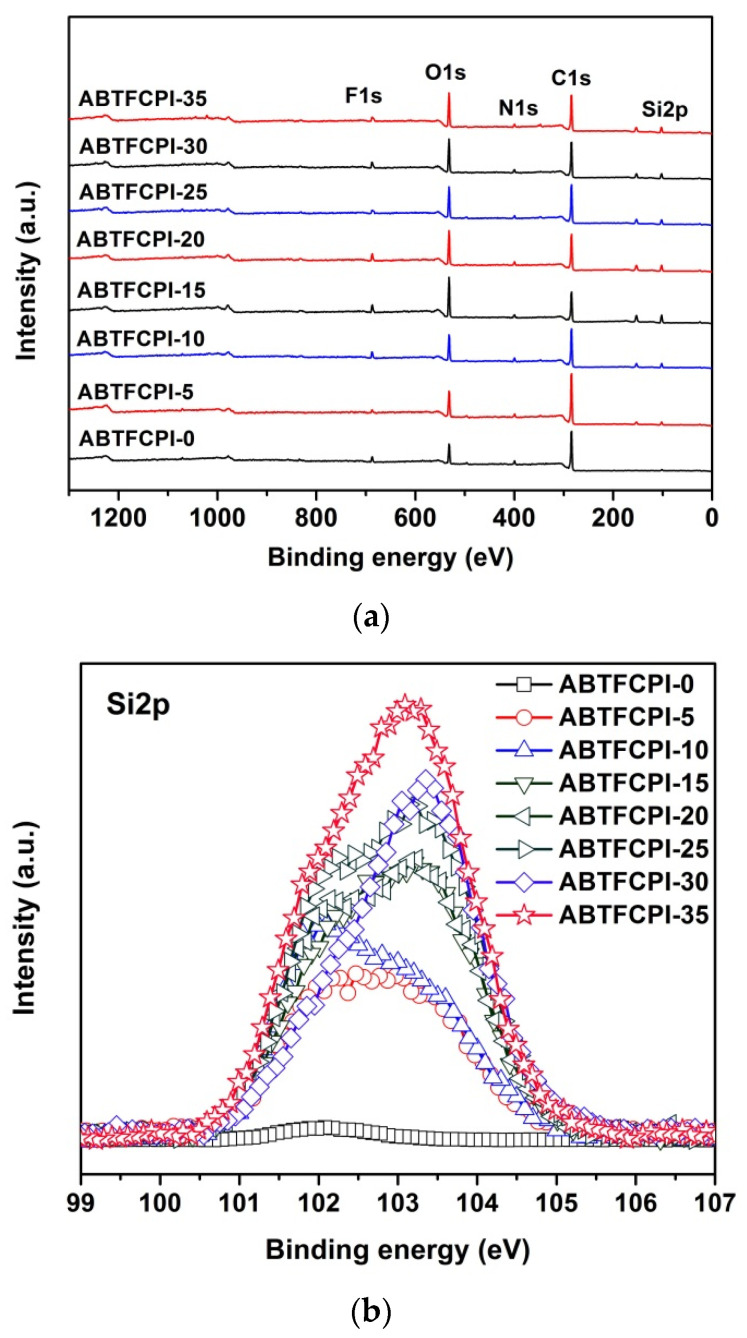
XPS plots of ABTFCPI films. (**a**) all-element plots; (**b**) Si2p plots.

Figure 5 depicts the XPS plots of the ABTFCPI films. All the samples showed clear absorptions of common elements, including the F1s at the binding energy of 688 eV, O1s at 532 eV, N1s at 400 eV, and C1s at 284 eV. However, only the composite films showed the absorptions of Si2p in the range of 100~153 eV, as can be seen in Figure 5a. In addition, from the expanded binding energy ranging from 99 to 107 eV (Figure 5b), one can observe that the absorption of Si2p increased with the increasing contents of the SiO_2_ fillers in the polymers. This is in good agreement with the composition characters of the nanocomposite films.

**Figure 6:**
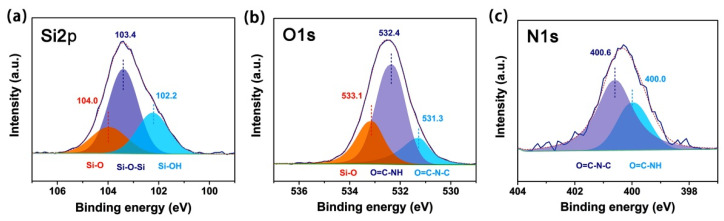
High-resolution XPS spectra of ABTFCPI-35 film. (**a**) Si2p; (**b**) O1s; (**c**) N1s.

Figure 6 further demonstrates the high-resolution spectra of Si2p, O1s, and N1s of the typical ABTFCPI-35 nanocomposite film. It can be seen from Figure 6a that the Si2p spectra exhibited three components with different relative intensities. The binding energy at 102.2 eV was due to the silicon of the Si-OH bond, the one at 104.0 eV was attributed to the Si-O bond, and the one at 103.4 eV was attributed to the Si-O-Si bond, respectively. It has been reported in the literature that the peaks of the Si-O-Si network structure in the SiO_2_ showed absorptions at 103.5 eV [34]. The current data have good consistency with the data reported in the literature. The O1s spectra of the ABTFCPI-35 film (Figure 6b) displayed three components, in which the binding energy at 531.3 eV was ascribed to the oxygen in the C=O bonds in the imide rings, the one at 532.4 eV was to the –CONH– bond. In addition, the binding energy at 533.1 eV was ascribed to the Si-O in SiO_2_, which was incorporated into this composite film. Figure 6c shows the N1s spectrum of the ABTFCPI-35 film. Two absorption peaks were observed at 400.6 eV and 400.0 eV, respectively. The former was due to the characteristic peak of nitrogen in the imide rings, and the latter was ascribed to the nitrogen in the amide units. Based on the data mentioned above, it could be deduced that the colloidal silica nanoparticles basically maintained the initial states in the nanocomposite films during the mechanical blending with the ABTFCPI matrix.

**Figure 7:**
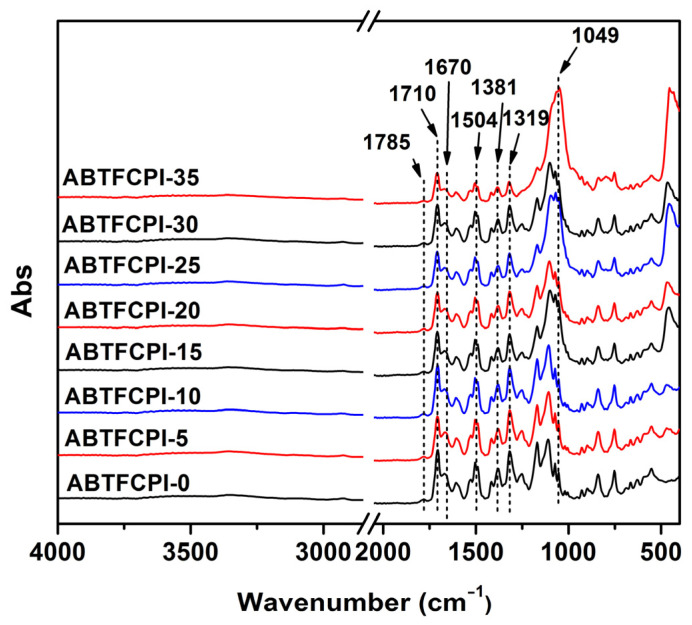
FTIR spectra of ABTFCPI films.

After confirming the successful incorporation of SiO_2_ nanoparticles into the ABTFCPI-0 matrix, the chemical structures of the polymers were identified by the FTIR measurements. Figure 7 shows the FTIR spectra of the ABTFCPI films together with the assignments of the main characteristic absorptions (Table 2). The spectra revealed the characteristic absorption peaks of the imide rings for all the polymers, including the asymmetrical and symmetrical carbonyl stretching vibrations at 1785 cm^−1^ and 1710 cm^−1^ and the C–N at 1381 cm^−1^. In addition, the phenyl C=C stretching vibrations at 1504 cm^−1^ and the amide (–CONH–) carbonyl stretching vibrations at 1670 cm^−1^ were also observed. The Si–O stretching vibrations at 1049 cm^−1^ were only detected for the composite films, indicating the incorporation of the silica fillers. At last, the C–H stretching vibrations in cyclohexane units at 3000~2900 cm^−1^ were also detected.

### 3.2. Optical Properties

Deterioration of the optical properties of the nanocomposite films due to the aggregation of the incorporated nanoparticles has been becoming one of the most concerning issues in the development of high-performance organic/inorganic composite optical films. After all, maintaining the intrinsic optical transparency and low color parameters of the pristine optical films is always the prerequisite in practical applications. Nano-sized silica fillers have been one of the most important functional fillers to compensate for the defects of the common polymeric films, such as the relatively poor dimensional stability at elevated temperatures, the low modulus, the low flame retardancy, and so on. However, the common silica nanoparticle is usually aggregated in the optical film matrixes, especially at high loading amounts. Thus, special surface treatments and additional dispersion treatments are usually required during the fabrication of the nanocomposite films. Even by these methodologies, the loading proportions of the nano-fillers in the final composite films were usually controlled at a relatively low level (<10 wt%). By contrast, colloidal silica (cSiO_2_) or silica sol nanoparticles dispersed in water or organic solvents exhibited good dispersion and distribution in the polymer films. Usually, the functionalization of the SiO_2_ surface introduces organic groups that make the colloidal SiO_2_ particles surface-active and sterically stable. The particles are amorphous, but the particle surface is composed of silanol groups, which is the hydroxyl group connected to the silicon atom. The increased surface activity of SiO_2_ particles enables them to act as an emulsifier. In general, silica sols are an aqueous suspension of colloidal SiO_2_ particles. In order to endow the silica sols with good compatibility with the ABTFCPI-0 matrix, the commercially available aqueous cSiO_2_ dispersion was modified by solvent replacement in the current work, in which the water was replaced by DMAc.

**Figure 8:**
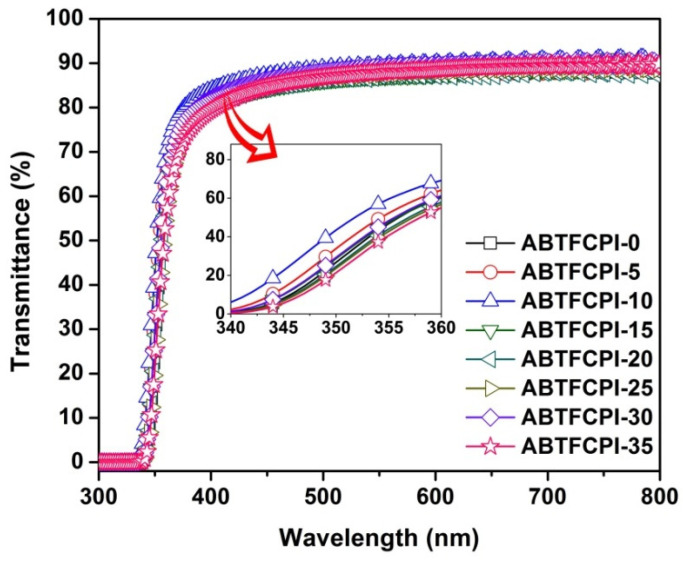
UV-Vis spectra of ABTFCPI films.

**Figure 9:**
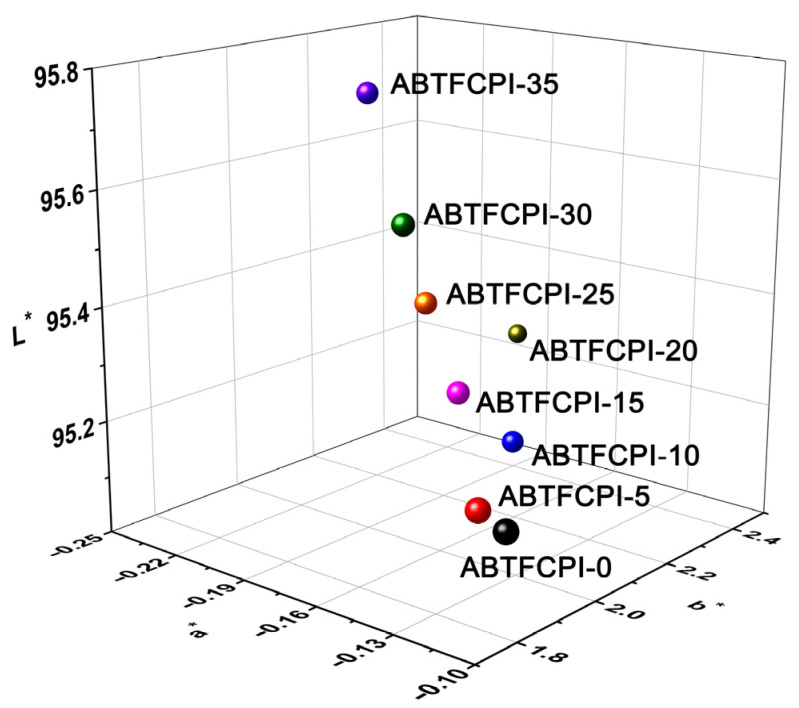
CIE Lab color parameters of ABTFCPI films.

Figure 8 and Figure 9 show the UV-Vis spectra and the 3D CIE Lab optical parameters of the ABTFCPI films, and the detailed data are summarized in Table 3. As expected, both the pristine ABTFCPI-0 and the nanocomposite films showed good optical transparency and low yellow indices. Incorporation of the cSiO_2_ nanoparticles did not deteriorate the optical properties of the derived films even at the high loading contents of 35 wt%. As shown in the figures and Table 2, the optical transmittance of the ABTFCPI films at the wavelength of 400 nm (T_400_) showed a trend of increasing first and then decreasing with the incorporation of silica nanoparticles. For example, the T_400_ value of the pristine ABTFCPI-0 film was 81.8%, while the ABTFCPI-5 film containing 5 wt% of silica fillers showed a value of 82.0%. As can be seen from the CIE Lab parameters shown in Figure 9, the brightness (L*) values of the nanocomposite films slightly increased with the increase of the silica fillers, but the yellow indices (b*) also increased, showing a trend of yellowing for the films. The haze values of the nanocomposite films remained at a relatively stable level. It can be concluded seen that the nanocomposite films could maintain the inherent optical characteristics of the pristine ABTFCPI-0 film when the silica contents were below 30 wt%.

### 3.3. Thermal Properties

The thermal properties of the ABTFCPI films, including the 5% weight loss temperatures (T_5%_), the residual weight ratios at 750 °C (R_w750_), the glass transition temperatures (T_g_), and the linear coefficients of thermal expansion (CTE) were investigated by TGA, DMA, and TMA measurements, respectively. The thermal properties data are tabulated in Table 3.

**Figure 10:**
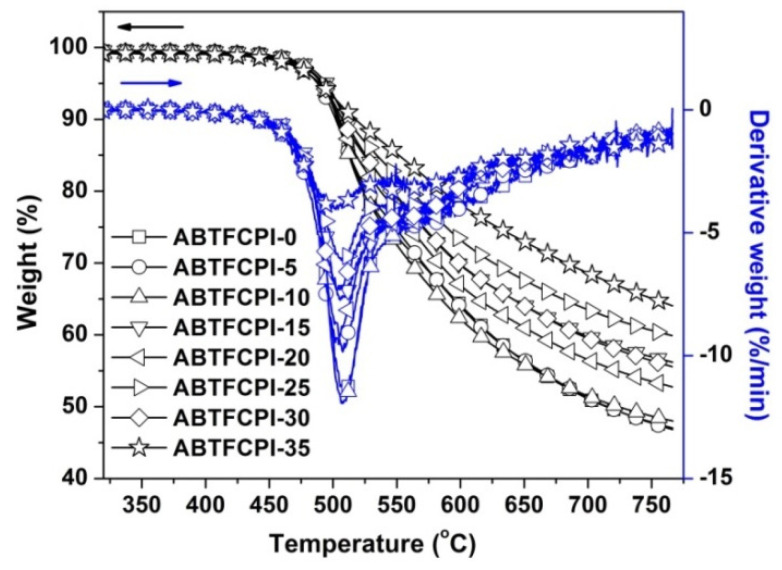
TGA and DTG curves of PI films in nitrogen.

First, the TGA and the derivative TG (DTG) plots of the samples are illustrated in Figure 10. All the ABTFCPI films showed good thermal stability before 450 °C. The films began to thermally decompose around 500 °C and revealed similar T_5%_ values in the range of 488~494 °C. Basically, the incorporation of the rigid-rod amide units in the semi-alicyclic CPIs efficiently increased the thermal resistance of the polymers due to the enhancement of the molecular chain interactions caused by the formation of strong hydrogen bonds [39]. According to the DTG plots, the most rapid thermal decomposition occurred in the range of 500~525 °C. At 750 °C, the samples maintained 47.8~64.7% of their original weights. From the above thermal data, it can be deduced that the incorporation of the cSiO_2_ nanoparticles had little effect on the initial thermal decomposition behaviors; however, it could efficiently increase the R_w750_ values of the composite films. This is mainly ascribed to the high thermal stability of the inorganic cSiO_2_ fillers at elevated temperatures.

**Figure 11:**
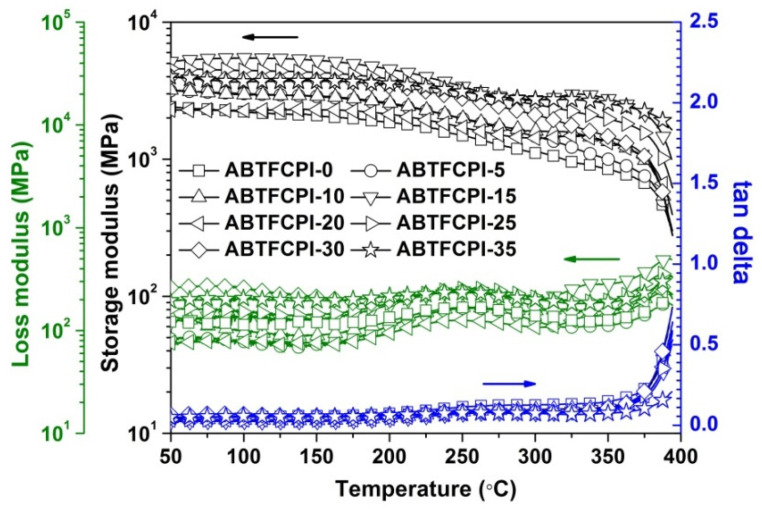
DMA curves of PI films.

Secondly, the T_g_ values of the ABTFCPI films were determined by the DMA measurements, as shown in Figure 11**.** It can be seen that the ABTFCPI films maintained most of the initial storage and loss modulus up to 350 °C, after which the modulus dramatically decreased. As for the tanδ plots, the inflection point temperatures of the samples were not detected before 400 °C, which is the upper limitation of the DMA equipment in the current work. The peak temperatures of the loss modulus plots were recognized as the T_g_ values of the films. According to this definition, the ABTFCPI-0 film showed a T_g_ of 390.3 °C, and the nanocomposite films exhibited similar T_g_ values, indicating the little effects of the cSiO_2_ nanoparticles on the glass transition behaviors of the films. This also verified the thermal stability of inorganic silica fillers at elevated temperatures. The high-T_g_ features of the current ABTFCPI films were mainly attributed to the rigid-rod benzanilide and biphenylene units in the polymers. Strong chemical bonds, such as hydrogen bonds, might form among the ABTFCPI molecular chains, which efficiently prohibited the free motions of the molecular segments in the polymers at elevated temperatures.

**Figure 12:**
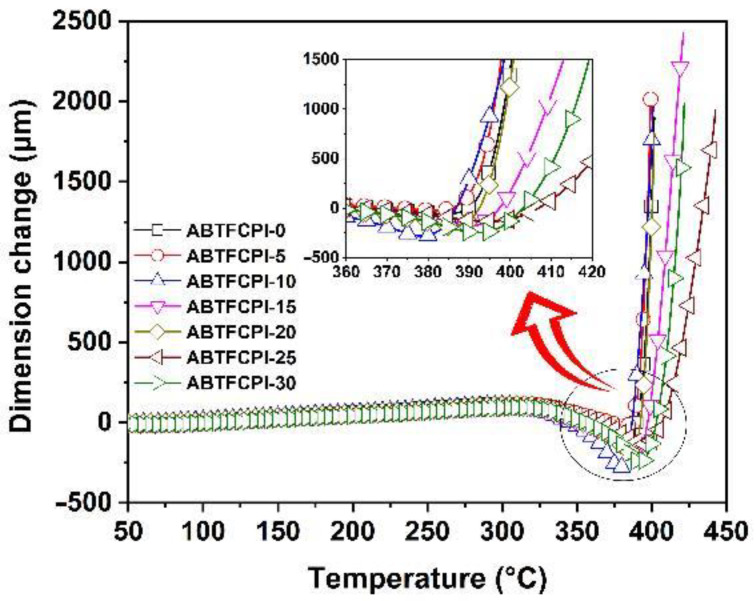
TMA curves of PI films.

At last, the high-temperature dimensional stabilities of the ABTFCPI films were evaluated by the TMA measurements. Figure 12 depicts the TMA curves of the ABTFCPI films together with the expanded image in the temperature range of 360~420 °C. The pristine ABTFCPI-0 film showed a CTE value of 32.7 × 10^−6^/K in the range of 50~250 °C (Table 3). This value ranked lower in all of the semi-alicyclic PI films, which are also the effects of the rigid-rod benzanilide and biphenylene units in the polymers. The incorporation of rigid amide or benzanilide linkages has been widely used to develop CPI films with low-CTE features. When incorporated with the cSiO_2_ nanoparticles, the CTE values of the CPI films further decreased. For example, the ABTFCPI-30 film showed a CTE value of 25.4 × 10^−6^/K, which was 22.3% lower than that of the pristine ABTFCPI film. In addition, it can be seen from the inserted picture that the ABTFCPI-25 and ABTFCPI-30 films with the higher loading contents of cSiO_2_ fillers showed much lower dimensional expansion at elevated temperatures. Undoubtedly, the inorganic cSiO_2_ fillers efficiently increased the dimensional stability of the nanocomposite films. On the one hand, this might be attributed to the good dispersion and distribution of the cSiO_2_ nanoparticles. On the other hand, the possible strong interactions between the hydroxyl (–OH) groups on the surface of the silica fillers and the amide bond (–CONH–) in the molecular structure of the ABTFCPI matrix film may also contribute to the reduction of CTE.

## 4. Conclusions

Semi-alicyclic CPI-SiO_2_ nanocomposite films with enhanced high-temperature dimensional stabilities were designed and prepared by incorporation of the colloidal silica nanoparticles into a CPI matrix film containing rigid-rod benzanilide and trifluoromethyl-substituted biphenylene units in the structure. The cSiO_2_ nanoparticles showed excellent compatibility with the CPI matrix with loading amounts over 30 wt%. The derived nanocomposite films exhibited excellent combined properties. The ABTFCPI-30 film showed the best properties, including a T_400_ value of 79.6%, a b* value of 2.15, a haze value of 0.50, a T_5%_ value of 491 °C, a T_g_ value of 386.1 °C, and a CTE value of 25.4 × 10^−6^/K, which makes it a good candidate for advanced optical applications.

## Figures and Tables

**Table 1:** Formulas for the preparation of ABTFCPI nanocomposite films.

Samples	ABTFCPI-0, DMAc (g, g)	cSiO_2_ (20 wt% in DMAc, g)	M_SiO_2__/M_total_ (wt%)
ABTFCPI-0	20.0, 80.0	0	0
ABTFCPI-5	19.0, 76.0	5.0	5
ABTFCPI-10	18.0, 72.0	10.0	10
ABTFCPI-15	17.0, 68.0	15.0	15
ABTFCPI-20	16.0, 64.0	20.0	20
ABTFCPI-25	15.0, 60.0	25.0	25
ABTFCPI-30	14.0, 56.0	30.0	30
ABTFCPI-35	13.0, 52.0	35.0	35

**Table 2:** Absorption peaks of ABTFCPI films.

Wavenumber (cm^−1^)	Assignment	Reference
1785	C=O symmetric stretching	[35]
1710	C=O asymmetric stretching	[35]
1670	amide C=O (amide I)	[36]
1504	C=C stretching	[35]
1381	C-N stretching	[35]
1319	C-F stretching	[37]
1049	Si-O stretching	[38]

**Table 3:** Optical and thermal properties of ABTFCPI films.

PI	Optical Properties ^a^						Thermal Properties ^b^			
	λ (nm)	T_400_ (%)	L*	a*	b*	haze (%)	T_g_ (°C)	T_5%_ (°C)	R_w750_ (%)	CTE (×10^−6^/K)
ABTFCPI-0	338	81.8	95.19	−0.10	1.77	0.25	390.3	492	47.8	32.7
ABTFCPI-5	336	82.0	95.21	−0.11	1.77	0.66	389.9	488	47.7	28.9
ABTFCPI-10	338	80.3	95.08	−0.18	2.33	0.24	387.2	491	48.7	28.1
ABTFCPI-15	337	80.4	95.24	−0.17	2.11	0.27	391.4	494	56.9	25.8
ABTFCPI-20	338	80.1	95.10	−0.25	2.82	0.54	389.2	492	53.5	27.5
ABTFCPI-25	333	83.8	95.54	−0.18	2.03	0.13	388.5	493	60.6	28.3
ABTFCPI-30	338	79.6	95.37	−0.19	2.15	0.50	386.1	491	56.4	25.4
ABTFCPI-35	339	77.2	95.70	−0.23	2.24	0.42	388.3	494	64.7	ND

^a^ λ: cutoff wavelength; T_400_: transmittance at the wavelength of 400 nm with a thickness of 50 μm; L*, a*, b*, see measurements part. ^b^ T_g_: glass transition temperatures defined as the peak temperatures of loss modulus in DMA measurements; T_5%_: temperatures at 5% weight loss; R_w700_: residual weight ratio at 750 °C in nitrogen; CTE: linear coefficient of thermal expansion in the range of 50~250 °C. ND: not detected.

## Data Availability

Data are contained within the article.
